# Low Dark-Current, High Current-Gain of PVK/ZnO Nanoparticles Composite-Based UV Photodetector by PN-Heterojunction Control

**DOI:** 10.3390/s16010074

**Published:** 2016-01-07

**Authors:** Sang-Won Lee, Seung-Hwan Cha, Kyung-Jae Choi, Byoung-Ho Kang, Jae-Sung Lee, Sae-Wan Kim, Ju-Seong Kim, Hyun-Min Jeong, Sai-Anand Gopalan, Dae-Hyuk Kwon, Shin-Won Kang

**Affiliations:** 1 School of Electronics Engineering, College of IT engineering, Kyungpook National University, 80 Daehakro, Bukgu, 41566 Daegu, Korea; sw3148@ee.knu.ac.kr (S.-W.L.); cha2155@naver.com (S.-H.C.); js1245@ee.knu.ac.kr (J.-S.L.); kei95304@gmail.com (S.-W.K.); jskim5772@ee.knu.ac.kr (J.-S.K.); hmjeong@ee.knu.ac.kr (H.-M.J.); gsai_anandh@yahoo.com (S.-A.G.); 2 Department of Sensor and Display Engineering, Kyungpook National University, 80 Daehakro, Bukgu, 41566 Daegu, Korea; dusen2147@naver.com; 3 Center for Functional Devices Fusion Platform, Kyungpook National University, 80 Daehakro, Bukgu, 41566 Daegu, Korea; bhkang@ee.knu.ac.kr; 4 Department of Electronics Engineering, Kyungil University, 50 Gamasil-Gil, Hayang-Eup, Gyeongbuk, 38428 Gyeongsan-Si, Korea; dhkwon@kiu.ac.kr

**Keywords:** UV photodetector, pn-heterojunction, hybrid photoactive layer, ZnO nanoparticles

## Abstract

We propose a solution-processable ultraviolet (UV) photodetector with a pn-heterojunction hybrid photoactive layer (HPL) that is composed of poly-n-vinylcarbazole (PVK) as a p-type polymer and ZnO nanoparticles (NPs) as an n-type metal oxide. To observe the effective photo-inducing ability of the UV photodetector, we analyzed the optical and electrical properties of HPL which is controlled by the doping concentration of n-type ZnO NPs in PVK matrix. Additionally, we confirmed that the optical properties of HPL dominantly depend on the ZnO NPs from the UV-vis absorption and the photoluminescence (PL) spectral measurements. This HPL can induce efficient charge transfer in the localized narrow pn-heterojunction domain and increases the photocurrent gain. It is essential that proper doping concentration of n-type ZnO NPs in polymer matrix is obtained to improve the performance of the UV photodetector. When the ZnO NPs are doped with the optimized concentration of 3.4 wt.%, the electrical properties of the photocurrent are significantly increased. The ratio of the photocurrent was approximately 10^3^ higher than that of the dark current.

## 1. Introduction

Ultraviolet (UV) photodetectors have a wide range of applications including environmental and biological research, and missile launches [1]. To date, most of the commercial photodetectors are fabricated by film growth from single-crystalline Si, SiC, and GaN which requires vacuum-processing [2,3,4,5]. However, this is needed not only in complex film growth for pn-junctions but also in very narrow interdigit texturing for efficient carrier transportation.

Recently, research of UV photodetectors based on hybrid material with organic-inorganic composites as a pn-heterojunction have attracted much attention due to efficient charge extraction which is attributed to the low binding energy between pn-heterojunctions and high photo sensitivity [6,7]. For example, Guo *et al.* fabricated a nanocomposite UV photodetector based on an interfacial trap-controlled charge injection by adding ZnO nanoparticles (NPs) to poly-n-vinylcarbazole (PVK) or Poly (3-hexylthiophene-2,5-diyl) (P3HT) [8]. These studies reported a new type of hybrid photodetector that has a Schottky contact in the dark and an ohmic contact under illumination. Wang *et al.* demonstrated a high-spectrum-selectivity hybrid UV photodetector by using electrodeposited ZnO nanorods and PVK as the electron acceptor and donor, respectively [9]. This photo-response of the photodetector showed a narrow band centered at 364 nm and a UV-visible on/off ratio of three orders of magnitude was also obtained at −5.0 V. In these reports, composite materials of the pn-heterojunction consist of conjugated polymer and metal oxide nanoparticles that have been focused on due to their physical, electrical, and optical properties. The PVK was used as the p-type polymer of which excellent photoconductive materials are made [10]. The ZnO NPs as an n-type metal oxide have a wide direct energy band gap of 3.4 eV and large exciton binding energies [11]. As mentioned before, when the ZnO NPs are embedded in polymers such as PVK and P3HT, exciton binding energy is reduced and photo-generated excitons can be efficiently separated to the adjacent layer. These reports showed not only improved photo-current gain but also a solution-process method for a UV photodetector. However, there was no consideration of optimized n-doping concentration in the pn-heterojunction hybrid photoactive layer (HPL), nor did the researchers discuss the optimized ratio of the pn-heterojunction which can induce efficient charge transfer.

In this study, to demonstrate effective charge transfer in a pn-heterojunction HPL, we demonstrated the effect of n-doping concentration in hybrid nanocomposites composed of p-type PVK and n-type ZnO NPs by analyzing the optical properties such as absorbance and photoluminescence (PL) according to variations of n-doping concentration by a previously reported method [12,13].

To the best of our knowledge, this is the first demonstration on the effect of device performance on absorbance and PL of HPL according to n-doping concentration. Thus, we fabricated the device of a UV photodetector using a simple solution method according to concentration of n-type ZnO NPs. Additionally, we confirmed the ratio of proper concentration at the pn-heterojunction for effective charge transfer. The optimized UV photodetector shows a significantly increased photocurrent when exposed to 1.0 mW of UV radiation.

## 2. Experimental Section

In our experiment, we used a hybrid composite of poly (n-vinylcarbazole) (PVK, Sigma-Aldrich, St. Louis, MO, USA) and ZnO NPs as a photoactive layer. To synthesize ZnO NPs, we used the optimized sol-gel method [14,15]. The 11.5 mmol zinc acetate dihydrate (Zn(CH_3_COO)_2_·2H_2_O, Sigma-Aldrich) was dissolved in 62.5 mL anhydrous methanol at 60 °C and followed by adding 14 mmol potassium hydroxide (KOH, Duksan Pure Chemicals Co., Ltd., Ansan-Si, Korea) to the 32.5 mL anhydrous methanol solution with gradual injection (1 mL/s). After two hours, the mixed solution became turbid and ZnO NPs were being grown. Then, the synthesized ZnO NPs were precipitated by centrifuging and were washed repeatedly with anhydrous methanol two times, and then finally dispersed in chlorobenzene, forming a transparent solution.

**Figure 1:**
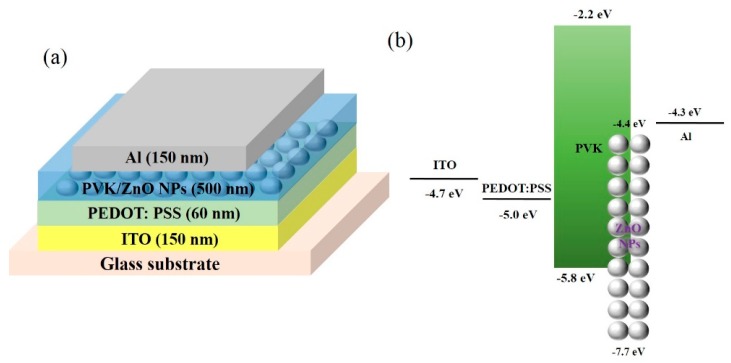
Schematic diagram of the fabricated UV photodetector based on the HPL of PVK/ZnO NPs: (**a**) device structure and (**b**) bandgap diagram.

We fabricated the solution-processible UV photodetector as a vertical structure device, which is composed of an anode, hole extraction layer (HEL), pn-heterojunction hybrid photoactive layer (HPL) and cathode. A schematic diagram and energy band diagram of the proposed UV photodetector are shown in Figure 1.

The Indium Tin Oxide (ITO) patterned glass substrate was cleaned ultrasonically in acetone, methanol and 2-propanol. The HEL of poly(ethy lene-dioxythiophene):polystyrenesulphonate (PEDOT:PSS, Clevios™ PVP AI 4083, Hanau, Germany) was spin-coated on a pre-treated ITO patterned glass substrate at 1500 rpm for 30 s and annealed at 150 °C on a hot plate for 10 min. We blended the PVK and ZnO NPs solution to form HPL. It is possible that PVK has good solubility to non-polar organic solvent such as chlorobenzene. Then, the ZnO NPs were doped into PVK matrix and the pn-heterojunction was formed between polymer and nanoparticles [8]. The HPL was deposited on the PEDOT:PSS film by the spin-coating method at 1500 rpm for 30 s, annealed at 110 °C for 30 min in a vacuum oven. The aluminum (Al) cathode was thermally deposited under high vacuum conditions. The photo-active area was 9 mm^2^.

In order to confirm the device performance, we measured the J-V characteristics under a UV light source which has a radiation power of 1.0 mW/cm^2^ in a 365 nm wavelength. The J-V data was measured by a computer-controlled voltmeter (Keithley 2400S, Beijing, China) and the LabVIEW program (National Instruments).

## 3. Results and Discussion

**Figure 2:**
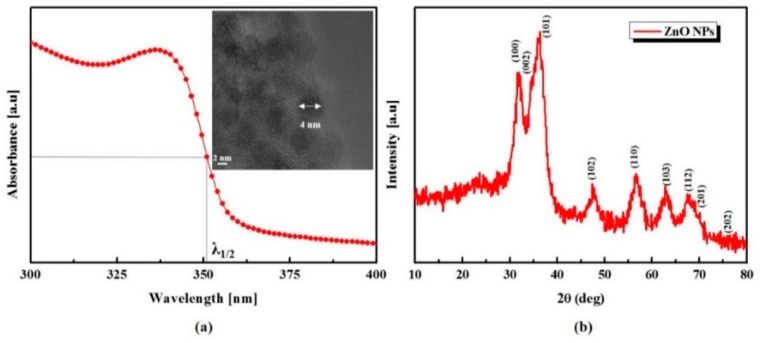
Characteristics of synthesized ZnO NPs: (**a**) UV-vis absorption spectrum and TEM image (inset); (**b**) XRD patterns for the ZnO NPs.

First of all, to confirm the optical property and size of ZnO NPs, we measured UV-visible absorption spectrum (UV-1601, Shimadzu, Kyoto, Japan). The particle growth can be easily defined by the UV-vis absorption spectrum as shown in the literature. In synthesized ZnO NPs, the absorption peak of 337 nm and the broad absorption band in the UV region are presented in Figure 2a. According to Meulenkamp, as is well known, the average size of synthesized nanoparticles was approximately 4 nm, which was calculated from λ1/2 (wavelength at which the absorption is 50% of that at the excitonic peak) of 351 nm [16]. To verify the crystallinity and shape, we analyzed the X-ray diffraction (XRD) and transmission electron microscopy (TEM). The observed size was 4 nm from the TEM image as shown in the Figure 2a inset and it is well matched with the calculated value from the UV-Vis spectrum. In Figure 2b, the XRD result shows the diffraction peaks and their relative intensities of spectrum all coincide with Joint Committee on Powder Diffraction Standards (JCPDS) card No. 36-1451. It exhibits the typical size-broadened reflections from wurtzite-structured ZnO NPs.

**Figure 3:**
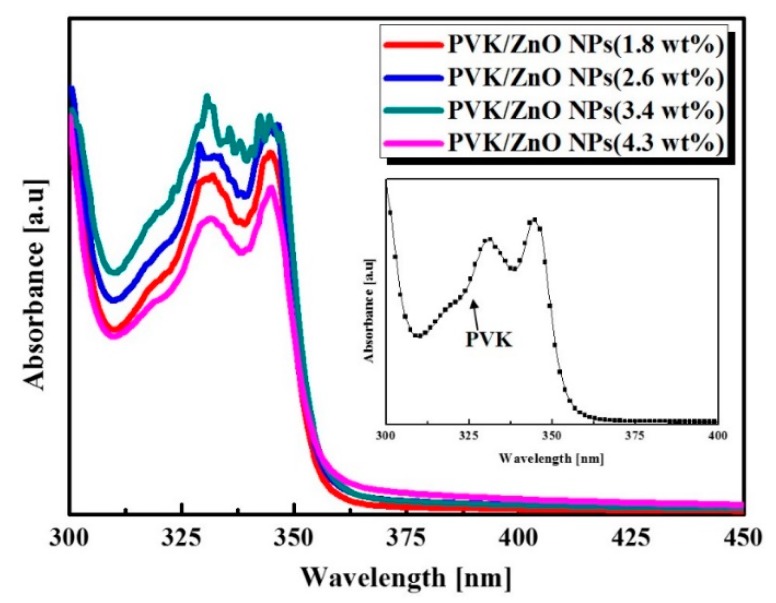
UV-vis absorption spectra of PVK/ZnO NPs hybrid composites and inset image is PVK optical properties.

**Figure 4:**
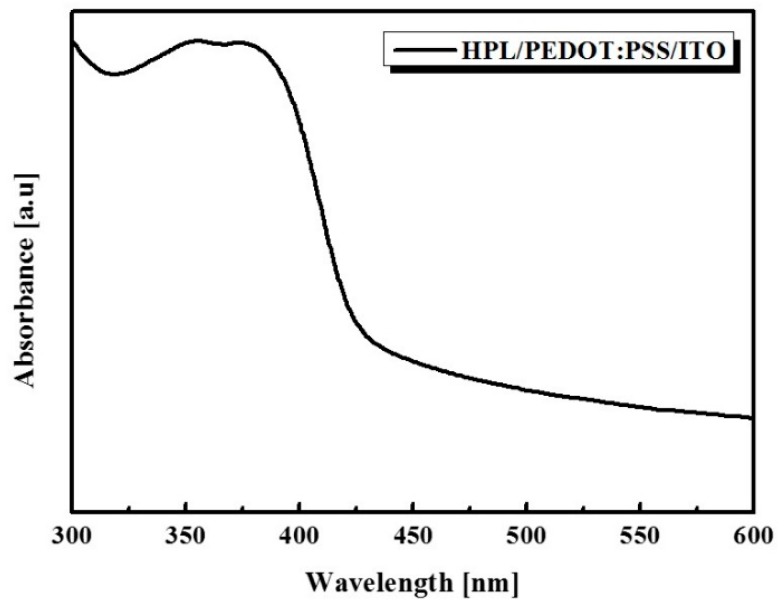
UV-vis absorption spectrum of HPL/PEDOT:PSS film.

To observe the effective photo-inducing ability of the HPL, we analyzed the UV-vis absorption spectra. As shown in Figure 3, the dominant absorption peak around 350 nm demonstrates that the proposed UV photodetector is able to generate photo-induced exciton by UV illumination. In order to verify the electrical and optical effect of the variation of n-type ZnO NPs doping concentration in the HPL, the PVK concentration was fixed at 2.0 wt.%. The ZnO NPs concentration was systemically varied from 1.8 wt.% to 4.3 wt.% in a PVK matrix. By comparing the absorption spectrum of each condition, the PVK/ZnO NPs hybrid composites doped with 3.4 wt.% of ZnO NPs showed superior absorbance than the PVK/ZnO NPs hybrid composites doped with l.8 wt.%, 2.6 wt.% of ZnO NPs. Results are derived from the charge transfer mechanism between PVK and ZnO NPs according to the proper concentration of the pn-heterojunction. These results indicated that the absorbance of hybrid composites increased as the ZnO NPs concentration increased, as shown in Figure 4. Also, the peak around 330 nm which originates from the PVK is increased in spite of the fixed PVK concentration. Thus, we confirmed that the absorbance of PVK/ZnO NPs hybrid composites dominantly depends on the ZnO NPs. However, the absorbance of PVK/ZnO NPs hybrid composites decreased when the ZnO NPs concentration reached 4.3 wt.%. When the concentration of ZnO NPs increases, the aggregation of ZnO NPs can be severely increased by the strong interaction among ZnO NPs [12,13] and the aggregation of ZnO NPs can be interrupted by efficient charge transfer between PVK and ZnO NPs.

Once again, to confirm the absorbance of HPL, we measured the absorbance for HPL/PEDOT:PSS film grown on the ITO substrate as shown in Figure 4. The absorbance spectrum of the ITO-deposited glass substrate is set as a reference background to remove the effect of the substrate. The dominant absorbance peak around 360 nm is attributed to the PVK in the HPL. Compared to the PVK in the solution state, the absorbance of the PVK in the thin film state is red-shifted. It results from the ordered structure of the polymer chain in PVK induced by the annealing process [17]. So, we use a 365 nm UV light source because of the dominated absorbance peak of the film from 300 to 400 nm.

**Figure 5:**
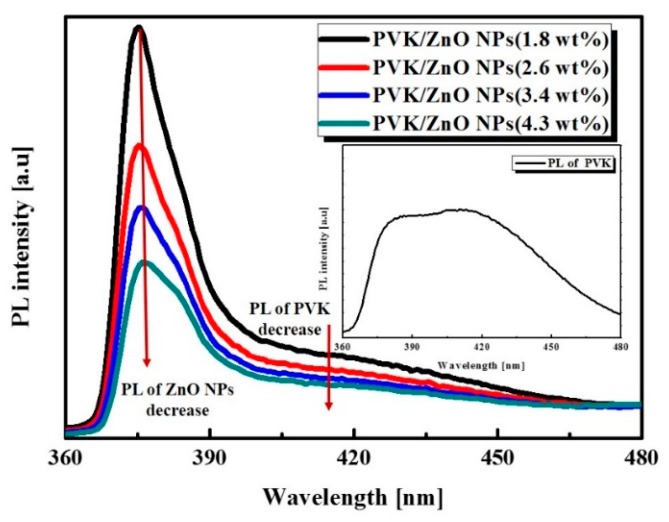
PL spectra of PVK /ZnO NPs hybrid composites and inset image is PL properties of PVK.

Figure 5 shows the PL spectra of the hybrid composites with 365 nm excitations and the inset image of Figure 5 is PL properties of PVK. The PL peaks of ZnO NPs and PVK are approximately 375 nm and 415 nm, respectively [18,19]. These results show a weakening of the PL intensity of the PVK corresponding to an increased concentration of ZnO NPs, in spite of the fixed PVK concentration. It results from the effective charge transfer from the PVK to ZnO NPs. Under UV excitation, weakened PL demonstrates reduced charge recombination because of the increased aggregation of ZnO NPs by strong inter-molecular electrostatic interactions.

**Figure 6:**
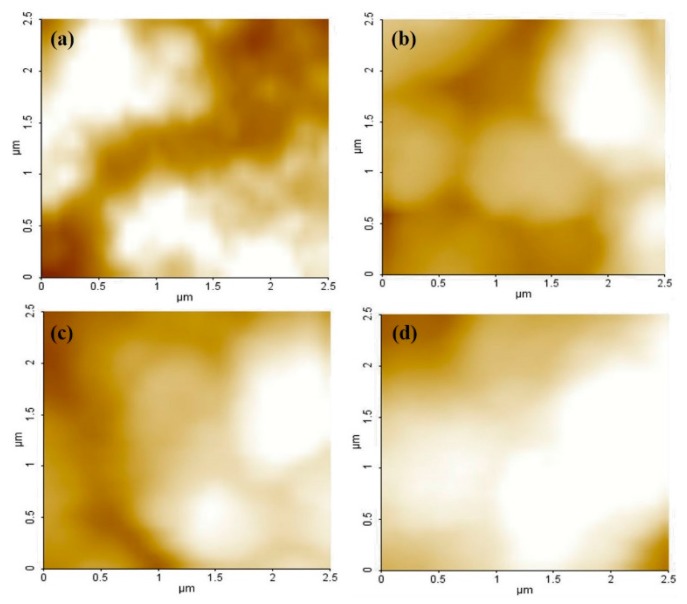
AFM image of HPL deposited on the PEDOT:PSS layer according to the concentration of ZnO NPs: (**a**) 1.8 wt.%; (**b**) 2.6 wt.%; (**c**) 3.4 wt.%; (**d**) 4.3 wt.%.

To confirm the aggregation phenomenon in HPL according to an increased concentration of the ZnO NPs, we analyzed the atomic force microscopy (AFM). As shown in Figure 6, the roughness of the HPL at ZnO NPs doping concentrations of 1.8 wt.%, 2.6 wt.%, 3.4 wt.% and 4.3 wt.% can be analyzed by the route mean square (RMS) R_q_ value of 89 nm, 493 nm, 555 nm and 718 nm, respectively. As the doping concentration of ZnO NPs in the PVK matrix increases, the morphology of the HPL is getting rougher due to the aggregation of ZnO NPs. When ZnO NPs are doped with more than 4.3 wt.%, the large clustered islands are formed by strong inter-molecular electrostatic interactions of ZnO NPs. This result shows that ZnO NPs are not uniformly dispersed in the PVK matrix and the HPL exhibited the large clustered islands on the surface. So, HPL via excess ZnO NPs can reduce the interface of PVK and ZnO NPs. As a result, high roughness leads to performance degradation in the devices.

**Figure 7:**
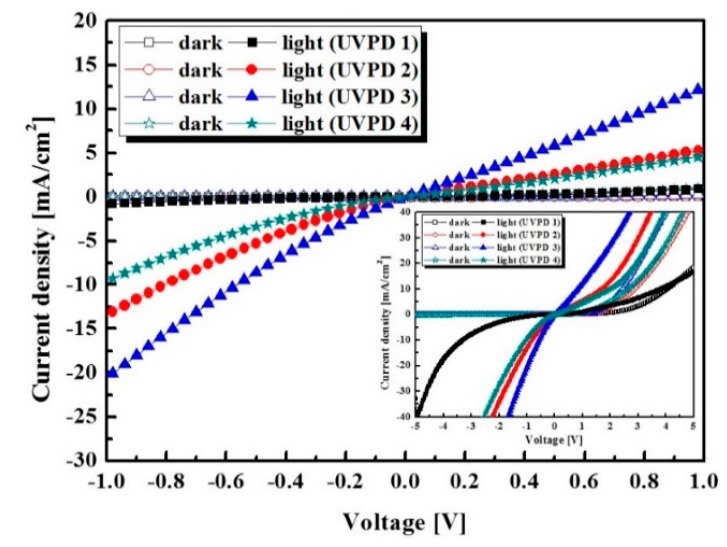
J-V characteristics of PVK/ZnO NPs hybrid UV photodetector according to concentration of ZnO NPs under UV illumination of 1 mW/cm^2^ and inset image shows J-V curve of pn-junctions in the UV photodetector.

In this way, to verify the electrical properties of the UV photodetector, we measured the J-V characteristics of the UV photodetector based on the PVK/ZnO NPs HPL according to the variation of the ZnO NPs concentration in the HPL with 365 nm UV illumination of 1 mW/cm^2^. Figure 7 and Table 1 shows J-V characteristics of the UV photodetector with various concentrations of 1.8 wt.% ZnO NPs (UVPD 1), 2.6 wt.% ZnO NPs (UVPD 2), 3.4 wt.% ZnO NPs (UVPD 3) and 4.3 wt.% ZnO NPs (UVPD4), respectively. The devices exhibit the dark current of 1.95 × 10^−3^ mA/cm^2^ (UVPD 1), 2.17 × 10^−3^ mA/cm^2^ (UVPD 2), 1.29 × 10^−3^ mA/cm^2^ (UVPD 3) and 8.09 × 10^−3^ mA/cm^2^ (UVPD 4) with a reverse bias of −0.5 V, respectively. The photocurrents of the devices are 0.23 mA/cm^2^, 5.28 mA/cm^2^, 8.58 mA/cm^2^ and 3.36 mA/cm^2^, respectively. The inset image of Figure 7 shows the J-V curve of the pn-junction in the UV photodetector. When the ZnO NPs are doped with a concentration of 3.4 wt.%, the photocurrent of UVPD 3 is significantly higher than the others. We also confirmed that the optimized condition of the n-doping concentration in HPL is 3.4 wt.%.

**Table 1:** Dark and photocurrent density of devices according to concentration of ZnO NPs.

	UVPD 1	UVPD 2	UVPD 3	UVPD 4
Dark current (mA/cm^2^)	1.95 × 10^−3^	2.17 × 10^−3^	1.29 × 10^−3^	8.09 × 10^−3^
Photocurrent (mA/cm^2^)	0.23	5.28	8.58	3.36

In other words, when the device is exposed to UV illumination, electrons and holes are generated at the interface of p-type and n-type materials due to the photovoltaic effect. The excited electrons and the remaining holes are swept in the anode and cathode, respectively. The electrons and holes diffuse to the n-type region and p-type region. So, when the concentration of the ZnO NPs is increased in the HPL, the performance of the devices can be improved because the proper ratio of the pn-heterojunction can induce efficient charge transfer in the device. However, when the ZnO NPs are doped with a concentration of 4.3 wt.%, the photocurrent of UVPD 4 is decreased compared to that of UVPD 3.

As shown in Figure 7, when ZnO NPs are doped with over than 4.3 wt.%, the performance of devices degrades due to the large clustered islands formed by strong inter-molecular electrostatic interactions. Therefore, by analyzing the AFM image, and optical and electrical properties of the hybrid composites, we confirmed that the charge transfer mechanism in the pn-heterojunction can be improved by controlling the concentration of ZnO NPs.

**Figure 8:**
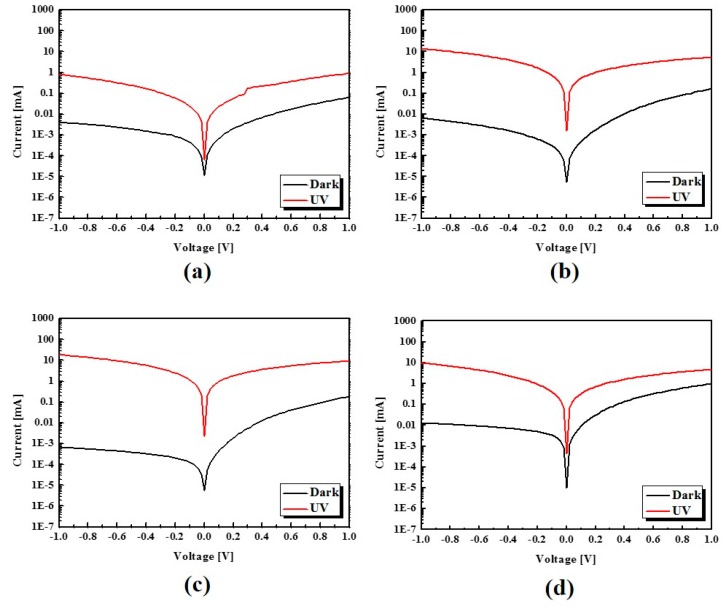
J-V characteristics of PVK/ZnO NPs hybrid UV photodetector under UV illumination of 1 mW/cm^2^: (**a**) UVPD 1; (**b**) UVPD 2; (**c**) UVPD 3; (**d**) UVPD 4.

The J-V characteristics shown in Figure 8 indicate the photocurrent of the device according to the concentration of ZnO NPs under illumination with 365 nm light. The ratio of photocurrent to the dark current was approximately about 10^3^ and the photocurrent of UVPD 3 doped with 3.4 wt.% ZnO NPs is higher than that of the others, and UVPD 4 doped 4.3 wt.% ZnO NPs exhibited a decreased ratio of photocurrent to dark current.

**Figure 9:**
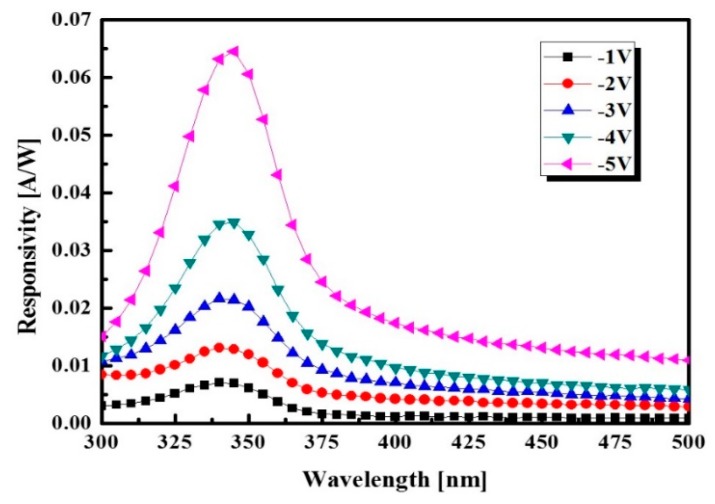
Spectral response of optimized UV photodetector as increased reverse bias.

In order to demonstrate the photo-response of the optimized UV photodetector, we measured the spectral response and photo-response speed. Under UV illumination of 1 mW/cm^2^, the fabricated device shows responsivity of 31 mA/W at a zero bias with a current density of 31 μA/cm^2^ and responsivity of 8.58 × 10^3^ mA/W at a reverse bias of −0.5 V with a current density of 8.58 mA/cm^2^. It is an extremely high responsivity of the UV photodetector based on HPL [20]. As shown in Figure 9, when the applied reverse bias increased from −1 V to −5 V, the spectral response was considerably increased to the short-wavelength region. The photo-response covers the short-wavelength of 320~375 nm.

**Figure 10:**
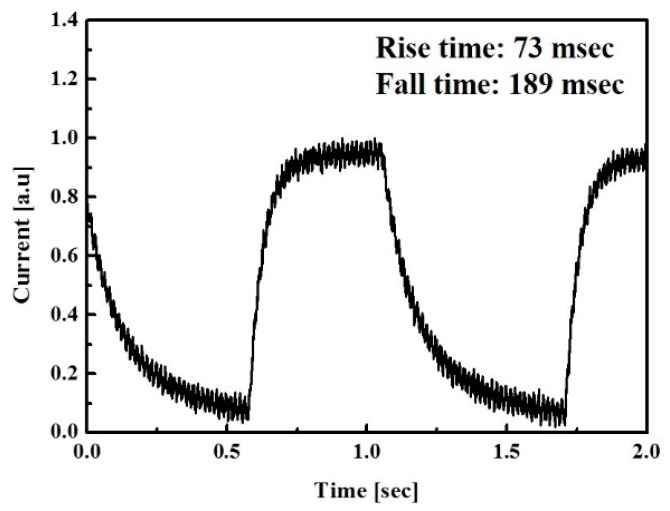
Transient photo response waveform of optimized UV photodetector with −1 V bias.

The photo-response speed was characterized by digitally controlled short light pulse. As shown in Figure 10, the transient photo current of the UV photodetector was measured under a bias of −1 V at a light intensity of 1 mW/cm^2^ from a 365 nm UV LED light source. The current rapidly increased under UV illumination and exponentially decreased without UV illumination. Additionally, the UV photodetector shows steady response with repetitive UV illumination. The transient time can be defined as rise time (photo current changes from 10% to 90% of the peak output value) and fall time (photo current decays from 90% to 10% of the peak output value) [8]. The results show a rise time of 73 msec and a fall time of 189 msec induced from efficient charge transfer in the pn-heterojunction between PVK and ZnO NPs.

As mentioned before, the proper ratio of the pn-heterojunction can induce efficient exciton diffusion from a PVK/ZnO NPs interface for effective charge transfer when the devices are exposed to UV light [21]. When the concentration of ZnO NPs is increased in the HPL, the performance of devices can be improved by controlling the concentration ratio of the pn-heterojunction. Additionally, the separated holes and electrons can be efficiently transferred through the anode and cathode. However, when the ZnO NPs in the pn-heterojunction were doped with more than 4.3 wt.%, the photocurrent of the devices decreased due to the extremely large clustered islands caused by ZnO NPs aggregations [12,22]. The excessively doped ZnO NPs can induce strong inter-molecular electrostatic interactions because the ZnO NPs are partially charged dielectric material. As a result, this aggregation in ZnO NPs reduces the absorbance of the HPL and interrupts the efficient charge transfer in the HPL, thus decreasing the photocurrent.

## 4. Conclusions

In summary, we fabricated the UV photodetector based on the HPL of the pn-heterojunction which is composed of PVK/ZnO NPs. The optical and electrical properties of HPL are determined by the concentration of doped ZnO NPs. When the ZnO NPs are doped with concentration of 3.4 wt.%, the photocurrent is significantly increased. Additionally, we confirmed the good properties of the doped 3.4 wt.% ZnO NPs. The proper ratio of the pn-heterojunction can be induced at the efficient charge transfer and a reduced recombination which resulted from controlling the concentration of ZnO NPs in a PVK matrix. Additionally, the photocurrent was significantly enhanced by efficient charge transfer in the pn-heterojunction between PVK and ZnO NPs. The photocurrent to dark current ratio of a fabricated hybrid UV photodetector was approximately 10^3^. Therefore, optical and electrical properties of HPL are highly dependent on n-type doping concentration and this study is very essential for UV photodetectors based on HPL.
